# Pre- and Post-COVID-19 Appraisal of Antimicrobial Susceptibility for Urinary Tract Infections at an Outpatient Setting of a Tertiary Care Hospital in Delhi

**DOI:** 10.7759/cureus.47095

**Published:** 2023-10-16

**Authors:** Suneeta Meena, Ginni Bharti, Purva Mathur

**Affiliations:** 1 Laboratory Medicine - Microbiology, All India Institute of Medical Sciences, New Delhi, IND; 2 Laboratory Medicine, All India Institute of Medical Sciences, New Delhi, IND; 3 Laboratory Medicine, Jai Prakash Narayan Apex Trauma Center, All India Institute of Medical Sciences, New Delhi, IND

**Keywords:** community, epidemiology, urinary tract infection, covid-19, antimicrobial resistance

## Abstract

Background: This study was conducted to understand the effect of the COVID-19 pandemic on urine culture and sensitivity results in an outpatient setting. There are plenty of data from inpatient and ICU settings but there is a paucity of data in outpatient or community settings. Thus, this study primarily targeted change in antibiotic resistance of urinary tract infection (UTI) agents in the pre- and post-COVID-19 period.

Methods: In the study, urine samples received in the Department of Laboratory Medicine (microbiology laboratory) with a preliminary diagnosis of UTI between April 2019 and March 2021 were analyzed. Urine cultures and antibiotic susceptibility tests of the patients included in the study were examined in two periods (pre-pandemic and post-pandemic).

Results: A total of 22,372 urine samples were received in the pre-pandemic period (April 2019 to March 2020) and 4885 samples in the post-pandemic period (April 2020 to March 2021). The positivity rate obtained from urine cultures sent post-COVID-19 pandemic (16%) was significantly higher than those sent before the COVID-19 pandemic (8%). According to cultures and antibiogram results, resistance to ampicillin, amikacin, ceftazidime (p < 0.05), co-trimoxazole, levofloxacin, gentamicin (p < 0.05), nitrofurantoin, fosfomycin, and tetracycline decreased compared with the pre-COVID-19 period.

Conclusions: In this study, we found that the frequency of significant bacteriuria increased significantly in the post-pandemic period. However, resistance to antibiotics decreased significantly in the post-COVID-19 period compared to the pre-COVID-19 period. There was no significant change in the etiology of UTI during the two time periods.

## Introduction

The SARS-CoV-2 pandemic, which started in Wuhan, China in November 2019, soon engulfed the whole world in its ambit. The first confirmed case of COVID-19 in India was identified in Kerala state on January 30, 2020 [1]. Since then, the country faced several waves and subsequent lockdowns to tackle the pandemic. Lockdown during the pandemic affected the prevalence of many infectious diseases. Several reports demonstrated how the COVID-19 pandemic influenced the epidemiology of many infections [2,3]. Infections that are transmitted through the same route as SARS-CoV-2 like other respiratory and gastrointestinal infections were expected to be decreased during the same pandemic. But, urinary tract infections (UTIs), which are not acquired through the same route, are not expected to be affected [4]. Attention of healthcare providers was diverted to COVID-19 care and outpatient services were the worst affected during the pandemic [5]. UTIs are the most common outpatient infections, with a lifetime incidence of 50−60% in adult women [6].

Thus, we aimed to evaluate the incidence of UTIs in an outpatient setting pre- and post-pandemic.

## Materials and methods

This was a retrospective observational study in which data from a laboratory providing services to outpatients were reviewed. It was conducted in the Division of Clinical Microbiology of Laboratory Medicine over two different time periods, i.e., the pre-pandemic period (April 2019 to March 2020) and the post-pandemic period (April 2020 to March 2021).

During each time period, the patient population comprised individuals of all age groups consecutively presenting to the outpatient clinic of our hospital with symptoms suggestive of UTI. Exclusion criteria were patients with negative urine cultures. Data analyzed were collected as a part of a study for which ethical approval was obtained from the Institutional Ethics Committee (Ref. No.: IECPG-761/30,01.2020).

Microbiological methods

Urine culture was performed using a semi‑quantitative technique on a cysteine lactose electrolyte‑deficient medium (CLED agar, HiMedia, Mumbai, India) [7]. Urine was cultured using a calibrated bacteriological 4 mm loop (0.05 ml) on CLED agar, and colonies were counted after overnight incubation at 37°C. Samples yielding significant bacteriuria (colony count > 105 CFU/ml) in the culture were further identified on the basis of gram stain, motility test, and routine biochemical reactions. Antibiotic sensitivity was put up by the Kirby-Bauer disc diffusion method on Mueller-Hinton agar (MHA) (HiMedia) adhering to the Clinical and Laboratory Standards Institute (CLSI) guidelines [8].

Statistical analysis

Data were compiled using Microsoft Excel 2019 (Microsoft Corporation, Redmond, WA). Quantitative variables were described as mean and standard deviation, and their comparison was performed using Student’s t-test. Qualitative variables were expressed as percentages, and their comparison was performed using the chi-square test and Fisher’s exact test. P-value < 0.05 was considered statistically significant.

## Results

A total of 22,372 urine samples were included during the first pre-pandemic period, of which 1899 urine specimens (8%) yielded significant growth. Whereas, during the second study period, 4885 samples were received, of which 782 (16%) yielded significant growth. The frequency of significant bacteriuria was significantly higher in the post-pandemic period (p < 0.05). Out of them, only patients with positive urine cultures were further included for analysis. Males were predominant during both periods. Age group analysis of only positive culture was done where no significant difference was seen, except for young adults (15-29 years), which were significantly higher during the post-pandemic period (Table 1).

**Table 1:** Demographic details of patients having significant bacteriuria during the two time periods

	Pre-pandemic period	Post-pandemic period	P-value
Total number of samples	22,372	4885	
Samples with significant bacteriuria	1899 (8%)	782 (16%)	<0.05
Age	40.19-25.4	38.5-21.21	
Male	56.7% (1080)	51.6% (408)	0.47
Female	42.8% (819)	48.4% (374)	
Age group			
<15	13.6 (260)	14 (110)	0.805
15-29	20.2 (385)	21.7 (170)	0.401
30-44	24.4 (465)	27.3 (214)	0.12
45-60	19.3 (367)	17.6 (138)	0.32
>60	22.2 (422)	19.2 (150)	0.087

The frequency of uropathogens during the two time periods was comparable between the two time periods (Table 2). *Escherichia coli* predominated during both the study periods and was significantly higher in the post-pandemic period (p = 0.046).

**Table 2:** Microbiological profile and frequency distribution of uropathogens during the two time periods

Microorganism	Pre-pandemic period (n = 1899)	Post-pandemic period (n = 782)	P-value
Escherichia coli	1006	447	0.049
Klebsiella spp.	326	130	0.77
Enterobacter spp.	4	2	0.1
Citrobacter spp.	19	4	
Proteus spp.	28	14	0.6
Morganella spp.	1	1	
Providencia spp.	1		
Pseudomonas spp.	246	71	0.0046
Staphylococcus spp.	124	72	0.0043
Enterococcus spp.	127	39	0.11
Acinetobacter	17	2	

The overall resistance to frequently used antibiotics is depicted in Table 3. The results revealed a statistically significant decrease in the resistance prevalence of isolated uropathogens to tested antibiotics in the post-pandemic period. This decreasing resistance pattern over a short time was seen for all antibiotics except amoxicillin-clavulanic acid (p = 0.0007), ampicillin-sulbactam (p = 0.0016), cefepime, and ciprofloxacin. The most significant decrease in resistance was seen for nitrofurantoin (p = 0.0001), followed by cotrimoxazole (p = 0.01). On comparing the resistance pattern among different groups of organisms, i.e., *Enterobacterales*, *Pseudomonas* species, and gram-positive organisms, a similar decrease in resistance pattern was seen between the two time periods. However, an increase in resistance was noticed for nitrofurantoin in *Enterobacterales* and a decrease in gram-positive organisms (Tables 4-6).

**Table 3:** Antimicrobial resistance pattern during the two study periods

Antibiotic	Pre-pandemic % (n = resistant isolates/total isolates)	Post-pandemic % (n = resistant isolates/total isolates)	P-value
Amoxicillin-clavulanic acid	70 (885/1264)	79.5 (257/323)	0.0007
Amikacin	38.9 (315/808)	28.5 (16/56)	0.1547
Ampicillin	88.5 (1220/1377)	84.7 (474/559)	0.0231
Ampicillin-sulbactam	40.2 (332/826)	48.8 (271/555)	0.0016
Ceftazidime	40 (88/220)	25 (8/32)	0.1209
Cefepime	67.3 (579/860)	70 (68/97)	0.6426
Cotrimoxazole	69 (996/1443)	58.8 (349/593)	0.001
Ciprofloxacin	70.4 (1236/1754)	73.4 (515/701)	0.1519
Levofloxacin	73.4 (856/1166)	68.1 (306/449)	0.035
Gentamicin	58.3 (94/161)	35.4 (22/62)	0.002
Nitrofurantoin	36.6 (360/982)	23.7 (146/614)	0.0001
Fosfomycin	13.1 (129/982)	12.3 (79/614)	0.87
Tetracycline	46 (41/89)	31.3 (16/51)	0.0866

**Table 4:** Antimicrobial resistance pattern of Enterobacterales during both the study periods

	Pre-pandemic	Post-pandemic	P-value
Amikacin	20	1.6	0.05
Ampicillin-sulbactam	22.2	44.9	0.002
Cefepime	32.5	4.8	0.51
Ciprofloxacin	43.6	73.5	0.23
Levofloxacin	22.5	40.1	0.0001
Nitrofurantoin	22.5	23.5	0.0001
Ampicillin	85.9	79.2	0.02
Amoxicillin-clavulanic acid	62.3	42.9	0.0004
Cotrimoxazole	65.2	54.3	0.00001
Fosfomycin	8.2	11.3	0.39

**Table 5:** Antimicrobial resistance pattern of Pseudomonas species during the study periods

Antimicrobial susceptibility testing	Pre-pandemic	Post-pandemic	P-value
Amikacin	12.7	8.4	0.72
Cefepime	49.5	54.9	0.04
Ceftazidime	36.8	11.2	0.1
Ciprofloxacin	44.4	47.8	0.49
Gentamicin	39.8	30.9	0.002
Levofloxacin	47	47.8	0.91
Piperacillin-tazobactam	8.4	28.1	0.006

**Table 6:** Antimicrobial resistance pattern of gram-positive organisms during the study periods

Antimicrobial susceptibility testing	Pre-pandemic	Post-pandemic	P-value
Ciprofloxacin	59.6	37.5	0.009
Levofloxacin	53.5	31.8	0.009
Nitrofurantoin	27.1	1.3	0.00046
Piperacillin-tazobactam	7	0	0.19
Fosfomycin	5.2	2.7	0.07
Tetracycline	10.5	11.1	0.28

## Discussion

The COVID-19 pandemic affected the epidemiology and transmission of several infectious diseases both in hospital and outpatient settings. Antimicrobial resistance is a silent epidemic that got a boost during the pandemic. There are several studies evaluating the impact of the pandemic on UTI and antimicrobial resistance [9-11]. With increased awareness about antimicrobial stewardship, most hospitals are shifting to the rational use of antibiotics and updating their antibiogram periodically [12]. But, in community settings, there is still a long way to go [13]. As the SARS-CoV-2 pandemic affected health care at all levels [14], this study made an attempt to study the effect of the pandemic on the UTI spectrum and antimicrobial resistance pattern during the pandemic.

Frequency of UTI

Although there was a significant decrease in the number of samples coming to the laboratory for testing, the frequency of UTI was significantly higher in the post-pandemic period. This could be attributed to the reduced number of samples coming to the laboratory during the post-pandemic period. Our results are in concordance with another study from a community setting [2]. However, according to other hospital-based studies, children requiring hospitalization due to UTI were not affected when compared to other diseases like respiratory tract infections, which are transmitted through the same route as SARS-CoV-2 [10]. Some studies evaluating the effect of the pandemic on UTI in community settings have shown decreased prevalence [15]. These differences in results can be possibly explained by outpatient settings where mixed groups (community and hospital) of patients are seen. Our study was a hospital-based study where urine culture results of outpatients were only processed and discharged patients from the hospital were also followed up. Although the same could not be analyzed separately in data. Moreover, during the pandemic, samples received for screening may have decreased like those of antenatal patients [16]. To conclude, some studies have shown a decreased incidence of UTI. In our study, the increased prevalence was seen, which could be attributed to our setting as explained.

Etiology

There was no change in the etiological spectrum during the two study periods (Table 2). *E. coli* predominated during both study periods and was significantly higher in the post-pandemic period. These results are in concordance with other community-based studies [17]. *E. coli* continues to be a cause of concern in the post-pandemic world as well. Therefore, it finds an exhaustive mention in the WHO priority list of antibiotic-resistant bacteria [18]. It has also been recognized as a pathogen of critical priority and a health challenge for the post-pandemic world [19].

Antimicrobial resistance

Antimicrobial resistance was a great cause of concern, especially in admitted COVID-19 patients acquiring secondary infection. Several studies reported increased antimicrobial resistance, especially in ICU settings [20]. Antimicrobial resistance escalated rapidly during the pandemic because of the increased and rapidly evolving resistance mechanisms of the pathogens to the commonly used or overused antibiotics. In UTIs, variable influences on antimicrobial resistance have been reported. In a study conducted in an emergency setting, a similar decrease in resistance has been reported [9]. This decrease in resistance over a short time period is a remarkable effect of the pandemic, which needs to be analyzed further. This decrease was significantly apparent for both oral and injectable antimicrobials except for amoxicillin-clavulanic acid, ampicillin-sulbactam, cefepime, and ciprofloxacin. However, resistance decreased significantly for many other antibiotics. This decrease was notable for nitrofurantoin (p = 0.0001), which is one of the recommended first-line agents for UTI [21]. Resistance also decreased to other antibiotics like amoxicillin-clavulanic acid, amikacin, and co-trimoxazole. These results are in concordance with another study conducted in an emergency setting [9]. There was no significant change in the resistance profile of fosfomycin. Fosfomycin has demonstrated excellent susceptibility against uropathogens in several studies [22-24].

There was no significant difference in the resistance profile of different groups of uropathogens, i.e., *Enterobacterales*, *Pseudomonas* species, and gram-positive organisms. However, an increase in resistance was noticed for nitrofurantoin in *Enterobacterales* but a decrease in gram-positive organisms. This could be attributed again to a decrease in the number of samples during the post-pandemic period.

Limitations of the study

Since this study was an analysis of routine laboratory work during pandemic times, some antimicrobials could not be reported because of supply chain disruption during lockdown. However, for the calculation of resistance, only those isolates were included whose sensitivity was available in the data, which has been clarified in Table 3. Also, this was primarily a laboratory-based study, so clinical data were not included in the data analysis.

A significant reduction in antimicrobial resistance was seen in a short time period. This finding needs to be further explored and analyzed, and lessons learned could have important public health implications. A closely resembling measure of "holiday antibiotic" practiced in several hospitals could be replicated to mitigate antimicrobial resistance.

## Conclusions

In conclusion, the frequency of UTIs post-pandemic increased but there was no significant change in the etiological spectrum of UTIs in our setting. However, resistance to antimicrobials decreased significantly during the post-pandemic period in all uropathogens in a short span of time.
